# A Prospective Noninterventional, Observational Study to Describe the Effectiveness and Safety of Trandolapril and Verapamil Single-Pill Combination in the Management of Patients with Hypertension and Type 2 Diabetes Mellitus: A Harvest TR Study

**DOI:** 10.1155/2020/2123601

**Published:** 2020-09-05

**Authors:** Enver Atalar, Fatih Eskin, Haci Bayram Tugtekin, Alpaslan Karabulut, Suleyman Kanyilmaz, Halil Kirbiyik, Ali Gokhan Ozyildiz

**Affiliations:** ^1^Hacettepe University Faculty of Medicine, Ankara, Turkey; ^2^Hitit University Corum Training and Research Hospital, Corum, Turkey; ^3^Umraniye Training and Research Hospital, Istanbul, Turkey; ^4^Beyhekim Public Hospital, Konya, Turkey; ^5^Balikesir Public Hospital, Balikesir, Turkey; ^6^Yildirim Beyazit University Yenimahalle Training and Research Hospital, Ankara, Turkey

## Abstract

Maintaining regular blood pressure control usually requires multidrug regimens rather than monotherapy. The objective of this study was to describe the effectiveness and safety of an angiotensin-converting enzyme inhibitor and a nondihydropyridine calcium channel blocker in a single-tablet combination in patients with hypertension, a heart rate higher than 70 beats/min, and type 2 diabetes mellitus (T2DM). This study was conducted in Turkey as a prospective, noninterventional, observational study. At 22 clinical sites, the data of 200 patients with hypertension were used for efficacy analysis; however, 262 patients received at least one dose of trandolapril/verapamil fixed-dose combination at two dose strengths. Systolic blood pressure (SBP), diastolic blood pressure (DBP), heart rate, PR interval, glycated haemoglobin (HbA1c), and albumin/creatinine ratios were recorded during 8 weeks of treatment. With treatment, the mean (±SD) SBP that was recorded as 162.8 (±14.642) mm Hg at baseline was reduced to 131.7 ± 11.1 mm Hg at week 8 (*p* < 0.05). Similarly, the mean DBP was reduced from 93.76 ± 9.16 mm Hg to 77.6 ± 7.6 mm Hg (*p* < 0.001). Following 8 weeks of treatment, SBP and DBP values were reduced below 140 mm Hg and 90 mm Hg in most patients (81.5%), respectively. The mean heart rate as evaluated using electrocardiography measurements was reduced to 78.25 beats/min at week 8 as compared with baseline during trandolapril/verapamil single-pill combination treatment (*p* < 0.001). Treatment with trandolapril and verapamil was well tolerated over 8 weeks with no unexpected safety signals. In conclusion, the single-pill combination of trandolapril and verapamil was considered effective in reducing and controlling blood pressure in patients with hypertension and T2DM. There was a significant improvement in HbA1c and ACR levels in a smaller subgroup of the patient cohort. The trandolapril/verapamil combination was evaluated as being safe and well-tolerated following a treatment period of 8 weeks. This trial was registered with NCT02298556.

## 1. Introduction

Hypertension has become a major preventable cause of cardiovascular disease (CVD) and all-cause mortality [1, 2]. The current 2018 ESC/ESH Guidelines for the management of arterial hypertension recommend a treatment strategy for lowering blood pressure (BP) below 140/90 mm Hg in all patients and when treatment is well tolerated, targeting 130/80 mm Hg or lower in most patients. Also, for patients aged younger than 65 years, it is recommended that systolic blood pressure (SBP) should be lowered to a range of 120-129 mm Hg [2, 3].

In patients with severe hypertension, prevention of cardiovascular risks becomes a priority and a combination treatment with different antihypertensive classes may provide better clinical outcomes but may have a negative impact on organ protection during long-term use [4]. In 2013, European Society of Hypertension- (ESH-) European Society of Cardiology (ESC) Hypertension Guidelines favoured the use of combinations of two different antihypertensive agents as a single-pill combination to improve adherence to treatment and to increase the rate of BP control and further endorsed by the current 2018 Guidelines [5, 6].

The positive antihypertensive effects of a combination product, ACEI with calcium channel blocker (CCB), were shown with superior metabolic effects and reflected in the ESC Guideline update much earlier, in 2009 [7], and 2018 ESC Guidelines recommended that the treatment of hypertension should be preferentially based on combinations of an ACEI or angiotensin receptor blocker (ARB) with a CCB and/or a thiazide/thiazide-like diuretic in a single pill to improve the efficiency and predictability of BP control. Trandolapril/verapamil slow release (SR) (2 mg/180 mg) is a more effective and well-tolerated treatment option for hypertension as compared with different monotherapy options and is as effective as fixed-dose combination therapies. The combination is better tolerated as compared with its active ingredients when administered as monotherapy agents [8, 9]. Furthermore, trandolapril/verapamil SR is an effective option for the treatment of essential hypertension in patients with coronary artery disease or type 2 diabetes mellitus (T2DM) [10, 11], providing cardiovascular homeostasis and lower adverse effects on the metabolism, making the treatment option suitable for use in diabetic populations [12].

The reduction of heart rate in patients with hypertension with increased heart rate was shown to exert a beneficial effect on cardiovascular complications and mortality, most notably in patients with coronary heart disease [13] as some studies revealed that a resting heart rate higher than 70 beats per minute in patients was a stand-alone risk factor with a higher risk of cardiovascular mortality [14]. In this noninterventional prospective study, the effectiveness and safety of an angiotensin-converting enzyme inhibitor (ACEI) and a nondihydropyridine calcium channel blocker (CCB) in a single-tablet combination were investigated in patients with hypertension and comparatively higher heart rates at baseline together with T2DM as a comorbidity condition.

## 2. Materials and Methods

### 2.1. Study Design

This was a prospective, noninterventional, multicentre, observational study conducted in Turkey in patients with hypertension who had elevated heart rates (70 beats/min or higher) and T2DM. The study was registered in a public repository before patient enrolment (ClinicalTrials.gov identifier: NCT02298556). After obtaining approval from the local ethics committee, local health authority approval was also obtained, and the study was conducted according to the principles of the Declaration of Helsinki. Due to the observational nature of the study, only patients who were previously initiated treatment with an ACEI and nondihydropyridine CCB single-pill combination, at least one week before enrolment, were included and BP measurements (home and physician's office) and electrocardiograms (ECG) were performed at baseline and during the 8-week follow-up period. All patients signed an informed consent form before enrolment.

### 2.2. Inclusion and Exclusion Criteria

**Figure 1:**
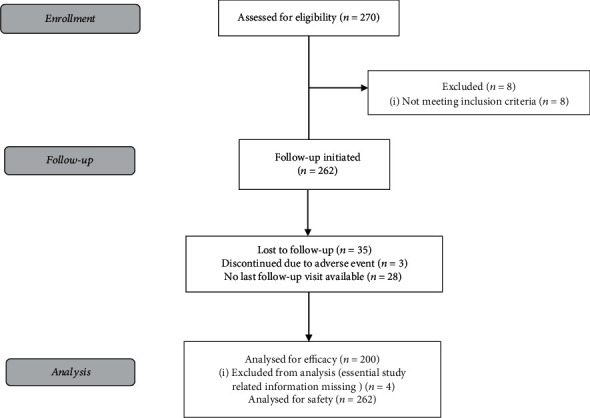
Consort diagram.

Eligible patients were aged ≥18 years and diagnosed as having hypertension based on systolic (SBP) or diastolic blood pressure (DBP) higher than 140 and/or 90 mm Hg at a screening visit. A total of 270 patients were enrolled in the study after the consenting process and were followed for 8 weeks. Data of 262 patients were evaluated; patients who did not meet the inclusion criteria were excluded from the effectiveness analysis by the independent expert panel. The primary endpoint was evaluated on the data of 200 patients, and safety analysis was performed on 262 patients. A consort diagram is given in Figure 1.

### 2.3. Assessment of Systolic Blood Pressure and Diastolic Blood Pressure

For monitoring of SBP, DBP, and heart rate, patients used automated BP-heart rate monitoring devices (OMRON M2, Omron Europe BV, Netherlands) and recorded their results in a patient diary. Office visit measurements were performed as two measurements in 10-minute ECG assessments performed for the measurement of heart rate and PR intervals at each visit, and two independent cardiology experts reviewed the ECGs and reported all abnormal clinical findings after the completion of the study.

### 2.4. Treatments

Two different dose strengths were prescribed: 2 mg trandolapril and 180 mg verapamil SR and 4 mg trandolapril and 240 mg verapamil SR depending on the baseline severity of hypertension. Switches between dose strengths were allowed.

### 2.5. Statistical Analysis

For the sample size calculation, a mean difference (standard deviation) of SBP difference between baseline and week 8 was hypothetically set, and based on this hypothesis, a sample size of 246 patients was found to be sufficient to produce a 95% confidence interval to detect paired mean differences with a margin of error of 2.5 mm Hg when the estimated standard deviation of the paired mean was 20 mm Hg.

The results were summarized by using descriptive statistics, and longitudinal data were analysed by using repeated measurement variance analysis and/or the paired-sample Student's *t*-test for dependent variables and continuous variables with normal distribution, and the Friedman test, Wilcoxon, and/or Mann-Whitney *U* test were used for variables with nonnormal distribution. Data of subgroups based on baseline blood pressure, sex, age, hypertension duration, T2DM duration, baseline body mass index (BMI), and baseline heart rate were compared.

## 3. Results

### 3.1. Demographics and Other Baseline Characteristics

The mean age of the patients was 55.73 ± 9.60 years, and female patients constituted 60.7% of the total population (*n* = 159). There were no significant differences in SBP and DBP at baseline between the sexes (*p* > 0.05). The mean SBP/DBP according to age, sex, hypertension severity, and BMI of the groups is given in Table 1. The duration of T2DM was not a predictive factor for BP levels. The majority of patients were recently diagnosed as having hypertension (diagnosis within 5 months, *n* = 192, 73.3%) and had a baseline heart rate of 85.74 beats/min (*n* = 262); there was no correlation between heart rate and SBP/DBP for the patients (*p* > 0.05).

### 3.2. Efficacy Results

**Figure 2:**
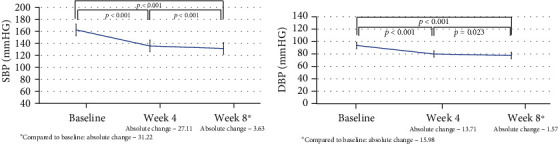
Absolute changes in systolic and diastolic blood pressure values during office measurements (all patients combined).

The primary objective of this noninterventional study was to describe the effectiveness of antihypertensive treatment with an ACEI and nondihydropyridine CCB single-pill combination in patients with heart rates higher than 70 beats/min and T2DM. With treatment, patients had a significant decrease in both SBP and DBP values; the mean SBP absolute change from baseline was calculated as -27.1 (+16.1) mm Hg after four weeks of treatment, and this reduction further increased to -31.2 (+15.5) mm Hg at the last visit (Figure 2). Absolute changes in DBP were -13.71 (+11.0) and -16.0 (+10.8) mm Hg at week 4 and week 8, respectively. All changes were statistically significant (*p* < 0.001).

Patients who responded to 2 mg trandolapril and 180 mg verapamil treatment were accepted as those who had a 20 mm Hg or greater reduction in SBP and/or 10 mm Hg or greater reduction in DBP. Depending on the BP evaluations during visits, patients were either switched to 4 mg trandolapril and 240 mg verapamil (higher dose combination) or received dose reductions between study visits, and almost all patients effectively responded to treatment.

**Figure 3:**
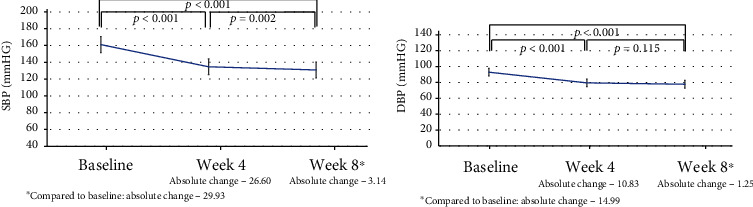
Absolute changes in systolic and diastolic blood pressure values during office measurements (2 mg trandolapril and 180 mg verapamil).

**Figure 4:**
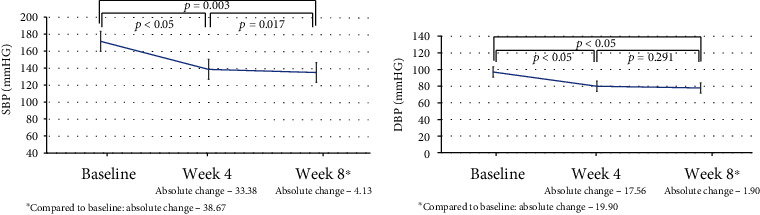
Absolute changes in systolic and diastolic blood pressure values during office measurements (4 mg trandolapril and 240 mg verapamil).

As expected, greater BP reductions were observed with the use of 4 mg trandolapril and 240 mg verapamil combination as the absolute change in 8 weeks reached -38.7 (+16.2) mm Hg for SBP and -19.9 (+11.5) mm Hg for DBP, all reductions being significant (Figures 3 and 4).

With the trandolapril-verapamil combination (2 mg/180 mg) treatment, a significant BP reduction was achieved in 74.6% of patients, and SBP continued to be lower at week 4 as compared with baseline. The percentage of patients with controlled BP increased to 82.0% during week 8. Similarly, low DBPs (≤90 mm Hg) were observed at 91.5% and 97.0% of the patient cohort at 4 and 8 weeks, respectively.

### 3.3. Assessment of Heart Rate, PR Interval, and Blood Pressure Relationship

**Figure 5:**
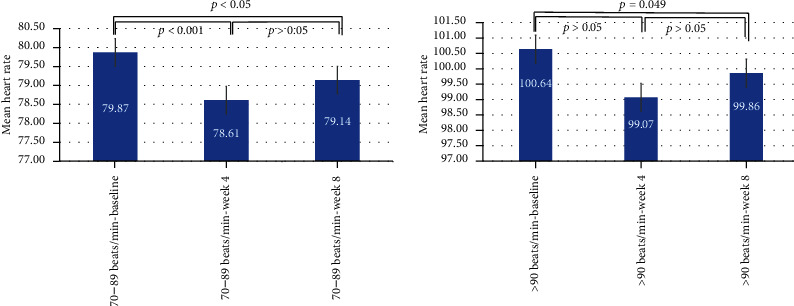
Heart rate—verified with ECG and measured with monitoring device.

The mean heart rate of patients was significantly reduced to 78.25 beats/min at week 8 with the treatment of trandolapril and verapamil single-pill combination during follow-up visits as compared with baseline (*p* < 0.001; Wilcoxon signed-ranks test) based on ECG measurement assessments. When patients were categorized into groups through initial heart rates (>70 and >90 beats/min), the absolute decrease in the heart rate was greatest for patients with higher baseline values (>90 beats/min) and was -5.50 (+10.9) after 8 weeks of treatment as compared with baseline (*p* = 0.049; Wilcoxon signed-ranks test). PR intervals were not altered by treatment (Figure 5).

### 3.4. Assessment of Glycated Haemoglobin (HbA1c) (%) and Albumin/Creatinine Ratio (ACR)

The evaluation of HbA1c (*n* = 164 at baseline, the number of patients decreased to 51 at week 8) and albumin/creatinine (*n* = 51) was performed in a comparatively limited number of patients due to the noninterventional nature of the study. The mean HbA1c level was 8.09% at baseline and decreased to 7.14% and 7.03% at the 4th and 8th-week visits, respectively. The mean ACR at week 4 was 45.75 mg/mmol and 39.18 mg/mmol at week 8 for 20 patients as compared with baseline (66.58 mg/mmol; *p* < 0.05), suggesting a positive outcome for patients with T2DM (Table 2).

### 3.5. Safety Evaluation

During the study, two serious adverse events (SAEs) were reported (0.4%) in one patient. A presyncope-like reaction occurred in this patient after the baseline visit, and the patient was subsequently admitted with bradycardia before the week 4 visit; the events resulted in treatment discontinuation. The patient was followed until complete resolution of the adverse events, and no other serious or nonserious adverse events occurred after treatment discontinuation.

There were five nonserious adverse events (NSAEs) observed in four patients (1.5%), which resulted in discontinuation of the 2 mg trandolapril and 180 mg verapamil or 4 mg trandolapril and 240 mg verapamil administration. The adverse events are presented in Table 3.

## 4. Discussion

In this noninterventional study, we investigated the effectiveness of a fixed-dose combination of the ACEI trandolapril and a CCB verapamil for the treatment of hypertension as effective BP control could not be achieved with a single agent. Some previous studies showed significant BP reductions with the same combinations such as the INVEST (INternational VErapamil SR/trandolapril STudy), which implemented this combination in an SR form showing that the combination was effective and well-tolerated [15] in achieving effective reductions in SNP and DBP and heart rate and with a positive outcome in HbA1c values.

This patient cohort was under treatment with other antihypertensive agents at the time of enrolment and despite treatment, still presented with high SBP (>140 mm Hg) at the outpatient visit and thus was asked to be enrolled to the study. With the trandolapril-verapamil combination treatment, a significant BP reduction was achieved in most patients, even after 4 weeks of treatment. The rate of patients with effective BP control was increased further at the end of the 8-week treatment period.

In a similar study conducted by Rubio-Guerra et al., the fixed-dose combination of trandolapril 2 mg and verapamil 180 mg was also found to be effective in the control of hypertension, with most patients having significant BP reductions and reaching the recommended therapeutic goals [16]. In the Tr/Ve study [17] conducted for the evaluation of the efficacy and safety of the verapamil SR and trandolapril combination, it was shown that the combination therapy in a single pill in the SR formulation was more effective than the use of the active ingredients of the combination and placebo in 631 patients with hypertension through 10 weeks of therapy. Our results also showed that the combination treatment at two different dose levels of trandolapril and verapamil showed similar results to the Tr/Ve study.

In our study, we planned to collect data on patients with T2DM and patients with a high ACR; however, the study was not successful in enrolling a sufficient number of patients with these comorbidities, so no conclusions could be reached as in the REGARDS study [17] due to the low number of patients with available values of HbA1c and ACR. Even though our data were limited, we observed statistically significant reductions in HbA1c and ACR values in patients receiving the study treatment. When compared with baseline, significant decreases in HbA1c and ACR levels were observed in our study, even after 4 weeks of treatment, suggesting a possible positive effect of the study treatment in patients with T2DM. On the other hand, previous studies such as the STAR trial [18] and BENEDICT trial [19] proved beneficial outcomes in patients with similar comorbidities such as T2DM or diminished renal function. The BENEDICT study indicated a significant decline in the risk of microalbuminuria in patients with hypertension and T2DM with normal albumin levels in the urine with long-term treatment of trandolapril plus verapamil and trandolapril alone. The STAR study was conducted with 240 patients with hypertension and impaired glucose tolerance and who were treated with a fix-dosed combination of trandolapril plus verapamil SR in comparison with losartan/hydrochlorothiazide. This was a long-term study, and the results showed that a fixed-dose combination of an ACEI with a nondihydropyridine CCB, in contrast to an ARB with a thiazide diuretic, achieved effective blood pressure goals and additionally avoided worsening of 2 h oral glucose tolerance test (OGTT) values in a cohort of patients with impaired glucose tolerance and metabolic syndrome. In this study, worsening of 2 h blood glucose levels in the losartan/hydrochlorothiazide group was paralleled by worsening of A1C and fasting glucose values at the study end. Although our results failed to provide conclusive outcomes in patients with T2DM due to the low number of patients with follow-up results, significant reductions were observed in HbA1c levels as well as ACR values.

The trandolapril/verapamil combination was also a well-tolerated treatment for our patient cohort; only a few adverse events were recorded. Various studies refer to constipation and cough as the more frequent adverse events; in our study, the most frequent nonserious adverse events were cough and pharyngitis (1.5% each). There was only one case of a serious adverse event, which occurred in one patient who experienced presyncope, followed by bradycardia two weeks after the initial reaction.

## 5. Conclusions

A single-pill combination of trandolapril and verapamil was found to be effective in controlling the BP of patients who were previously diagnosed as having hypertension and T2DM, but not efficiently treated with a single antihypertensive medication. The fixed-dose combination of trandolapril and verapamil may also have a positive effect on patients' diabetic status and renal function preservation in terms of HbA1c and ACR levels, in line with the results observed with this noninterventional study. Thus, the fixed-dose trandolapril and verapamil combination was considered safe and well-tolerated in patients with hypertension and diabetes following 8 weeks of treatment.

## Figures and Tables

**Table 1:** Mean blood pressure values at baseline according to the sex, age groups, BMI, and hypertension classification.

	SBP (mm Hg)			DBP (mm Hg)		
Sex	Male	Female		Male	Female	
*n* (%)^a^	105 (38.9%)	165 (61.1%)		105 (38.9%)	165 (61.1%)	
Mean (±SD)	163.37 (14.51)	162.35 (14.39)		94.78 (8.22)	93.10 (9.69)	
Age groups	<50 years	51-60 years	>60 years	<50 years	51-60 years	>60 years
*n* (%)^b^	81 (30.9%)	96 (36.6%)	85 (32.4%)	81 (30.9%)	96 (36.6%)	85 (32.4%)
Mean (±SD)	162.89 (14.28)	163.07 (14.47)	162.25 (14.66)	94.96^c^ (10.02)	94.43 (8.74)	91.86^b^ (8.54)
BMI (kg/m^2^)	18.5-24.9	25-30	≥30	18.5-24.9	25-30	≥30
*n* (%)^b^	18 (6.9%)	95 (36.3%)	149 (56.9%)	18 (6.9%)	95 (36.3%)	149 (56.9%)
Mean ^d^ (±SD)	168.06 (17.20)	161.85 (14.38)	162.68 (14.05)	92.83 (9.13)	93.38 (7.44)	94.11 (10.14)
	SBP (mm Hg)			DBP (mm Hg)		
Hypertension classification	Grade 1	Grade 2	Grade 3	Grade 1	Grade 2	Grade 3
*n* (%)^b^	96 (36.6%)	123 (46.9%)	38 (14.5%)	139 (53.0%)	49 (18.7%)	18 (6.8%)
Mean (±SD)	149.86 (5.33)	166.04 (5.66)	188.21 (7.43)	92.79 (2.88)	101.61 (2.27)	114.22 (6.47)

^a^Males and females were compared within the SBP and DBP groups; *p* > 0.05; Mann-Whitney *U* test (*N* = 270). ^b^Percentages were calculated according to the total patient number (*n* = 262). ^c^Patients with age < 50 years vs. patients with age > 60 years; *p* = 0.008; Mann-Whitney *U* test. ^d^Percentages were calculated according to the total number of patients (*n* = 262), and the mean values were compared with the SBP and DBP groups; *p* > 0.05; Kruskal-Wallis test.

**Table 2:** HbA1c levels and albumin/creatinine ratio.

	HbA1c levels		
	Baseline (%) (*n* = 164)	Week 4 (%) (*n* = 61)	Week 8 (%) (*n* = 51)
Mean (±SD)	8.09 (1.77)	7.14^a^ (1.29)	7.03^b^ (1.06)

	Albumin/Creatinine ratio (mg/mmol)		
	Baseline (*n* = 20)	Week 4 (*n* = 20)	Week 8 (*n* = 20)
Mean (±SD)	66.58^c,d^ (101.64)	45.75^e^ (64.64)	39.18^e^ (60.90)

^a^Compared with baseline vs. week 4; *p* < 0.001; Wilcoxon signed-rank test. ^b^Compared with baseline vs. week 8; *p* < 0.001; Wilcoxon signed-rank test. ^c^Compared with baseline vs. week 4; *p* < 0.05; Wilcoxon signed-rank test. ^d^Compared with baseline vs. week 8; *p* < 0.05; Wilcoxon signed-rank test. ^e^Compared with week 4 vs. week 8; *p* < 0.05; Wilcoxon signed-rank test.

**Table 3:** Summary list of serious adverse events and nonserious adverse events.

Serious adverse events	*n* (%)
Primary system organ class Preferred term [*n* (%)]	*N* = 270
Any class	1 (0.37%)
Cardiac disorders	1 (0.37%)
Bradycardia	1 (0.37%)
Nervous system disorders	1 (0.37%)
Presyncope	1 (0.37%)

Nonserious adverse events	
Primary system organ class Preferred term [*n* (%)]	*N* = 270
Any class	4 (1.48%)
Respiratory, thoracic, and mediastinal disorders	4 (1.48%)
Cough	4 (1.48%)
Infections and infestations	1 (0.37%)
Pharyngitis	1 (0.37%)

AE: adverse event; SOC: system organ class, PT: preferred term MedDRA 20.0; *N* = number of patients who received 2 mg trandolapril and 180 mg verapamil or 4 mg trandolapril and 240 mg verapamil; *n* (%) = number and%of patients with at least one SAE.

## Data Availability

The data used to support the findings of this study are available from the corresponding author upon request.
